# Austro-Asiatic Tribes of Northeast India Provide Hitherto Missing Genetic Link between South and Southeast Asia

**DOI:** 10.1371/journal.pone.0001141

**Published:** 2007-11-07

**Authors:** B. Mohan Reddy, B. T. Langstieh, Vikrant Kumar, T. Nagaraja, A. N. S. Reddy, Aruna Meka, A. G. Reddy, K. Thangaraj, Lalji Singh

**Affiliations:** 1 Biological Anthropology Unit, Indian Statistical Institute, Hyderabad, India; 2 Department of Anthropology, Northeast Hill University, Shillong, India; 3 Genome Institute of Singapore, Singapore, Singapore; 4 Centre for Cellular and Molecular Biology, Hyderabad, India; University of Montreal, Canada

## Abstract

Northeast India, the only region which currently forms a land bridge between the Indian subcontinent and Southeast Asia, has been proposed as an important corridor for the initial peopling of East Asia. Given that the Austro-Asiatic linguistic family is considered to be the oldest and spoken by certain tribes in India, Northeast India and entire Southeast Asia, we expect that populations of this family from Northeast India should provide the signatures of genetic link between Indian and Southeast Asian populations. In order to test this hypothesis, we analyzed mtDNA and Y-Chromosome SNP and STR data of the eight groups of the Austro-Asiatic Khasi from Northeast India and the neighboring Garo and compared with that of other relevant Asian populations. The results suggest that the Austro-Asiatic Khasi tribes of Northeast India represent a genetic continuity between the populations of South and Southeast Asia, thereby advocating that northeast India could have been a major corridor for the movement of populations from India to East/Southeast Asia.

## Introduction

Two major routes have been proposed for the initial peopling of East Asia; one via Central Asia to Northeast Asia, which subsequently expanded towards Southeast Asia and beyond, and the other through India to Southeast Asia and further to different regions of East Asia [1]. It is pertinent in this context that the Indian subcontinent has been considered as a major corridor for the migration of human populations to East Asia [2]–[4]. Given its unique geographic position, Northeast India is the only region which currently forms a land bridge between the Indian subcontinent and Southeast Asia, hence hypothesized as an important passage for the initial peopling of East Asia. This region is inhabited by populations belonging to Indo-European, Tibeto-Burman and Austro-Asiatic linguistic families. Whereas Indo-European populations are also found in other parts of India, West Asia and Europe but absent in East Asia, Tibeto-Burman populations are otherwise found only in East Asia. However, Austro-Asiatic speakers, hypothesized as probably the earliest settlers in the Indian subcontinent ([5] and references their in), are also found in other parts of India as well as in East/Southeast Asia. Therefore, if Northeast India had served as an initial corridor, it is likely that the Austro-Asiatic tribes of this region should provide hitherto missing genetic link, which may reflect genetic continuity between Indian and East/Southeast Asian populations. Based on mitochondrial DNA (mtDNA) and Y-chromosome markers, Cordaux et al. [6] observed genetic discontinuity between the Indian and southeast Asian populations and inferred that Northeast India might have acted as a barrier rather than the facilitator of the movement of populations both into and out of India. However, this study included only a few Tibeto-Burman populations of Northeast India whose distribution is restricted only to this region in India [7]–[8], besides a few other populations from other parts of India, possibly with no genetic link with East Asians. It is therefore imperative that the framework of testing such a hypothesis should include adequate representation of these people from Northeast India. Further evidence is needed by way of determining the mtDNA and Y-chromosome haplogroups/lineages of the Austro-Asiatic tribes of the northeastern region and their comparison with appropriate set of South and Southeast Asian populations.

**Figure 1:**
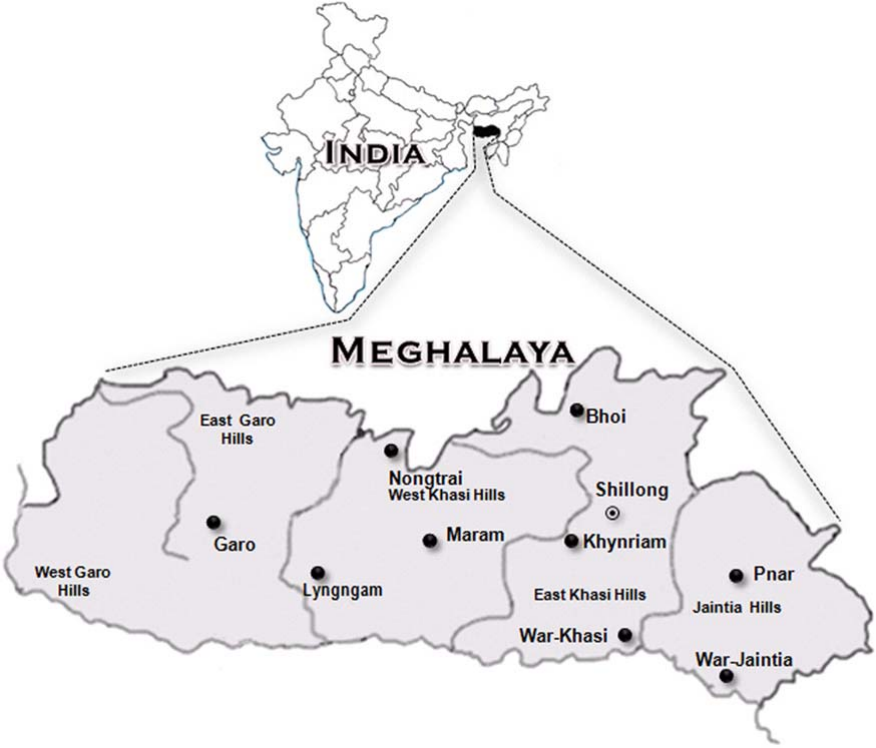
Map of Meghlaya showing the core areas of geographic distribution of the Khasi and Garo populations.

The Northeastern part of India is sandwiched by the marked presence of the young fold mountains of Eastern Himalayas on the northern side and the Indian Ocean on its southern side. The archaeological evidences, based on stone tools from the Garo hills of the Meghalaya region of Northeast India (Figure 1), suggest that this region might have been inhabited as early as in the Paleolithic period [9]–[12]. Within this Meghalaya region, one finds Khasi tribes whose language belongs to the Khasi-Khmic subfamily of the Austro-Asiatics [13], the other two branches of Austro-Asiatics being Mundari and Mon-Khmer, amidst the ethnic majority of Tibeto-Burman populations. Further, the Austro-Asiatic populations of Meghalaya and one of the contiguous Tibeto-Burman tribes namely, Garo, practice matrilineal pattern of descent and matrilocal pattern of residence while the other populations of this region are patrilineal and patrilocal. Such extreme cultural and linguistic diversity of this region may also imply high degree of genetic heterogeneity possibly due to passage of diverse populations through this region. Despite a possible major role played in the population dispersal by the Northeastern region, as transect between India and East Asia, the extent and nature of mtDNA and Y-chromosome diversity of this region is not adequately studied. Therefore, we present results based on the analyses of mtDNA and Y-Chromosome Single Nucleotide Polymorphisms (SNPs) and Short Tandem Repeats (STRs) data of the 8 subgroups of the Austro-Asiatic Khasi from Northeast India (Table 1 and Figure 1), probably for the first time, along with that of the other relevant populations to explore the missing genetic link between the Indian and Southeast Asian populations.

**Table 1:** Areas of sampling and the linguistic affiliations of the Meghalayan populations along with the number of samples typed for Y-chromosome and mtDNA.

Sl. No.	Name of the Populations	Traditional Occupation & Area of Sampling (at district level)		Sample Size	
				Y	mtDNA
1	Bhoi	SC	Ri-Bhoi Dt.	32	29
2	Maram	SA	West Khasi Hills	64	60
3	Lyngngam	SC	West Khasi Hills	60	74
4	Nongtrai	SC	West Khasi Hills	18	27
5	War-Jaintia	HC	Jaintia Hills	19	17
6	War-Khasi	HC	East Khasi Hills	29	29
7	Pnar	SA	Jaintia Hills	44	50
8	Khynriam	SA	East Khasi Hills	87	82
9	Garo	SC & SA	South Garo Hills & Others	71	76
	Total			424	444

SC: Shifting Cultivators; SA: Settled Agriculturists; HC: Horticulturist

## Results

### Distribution and diversity of Y-chromosome

**Figure 2:**
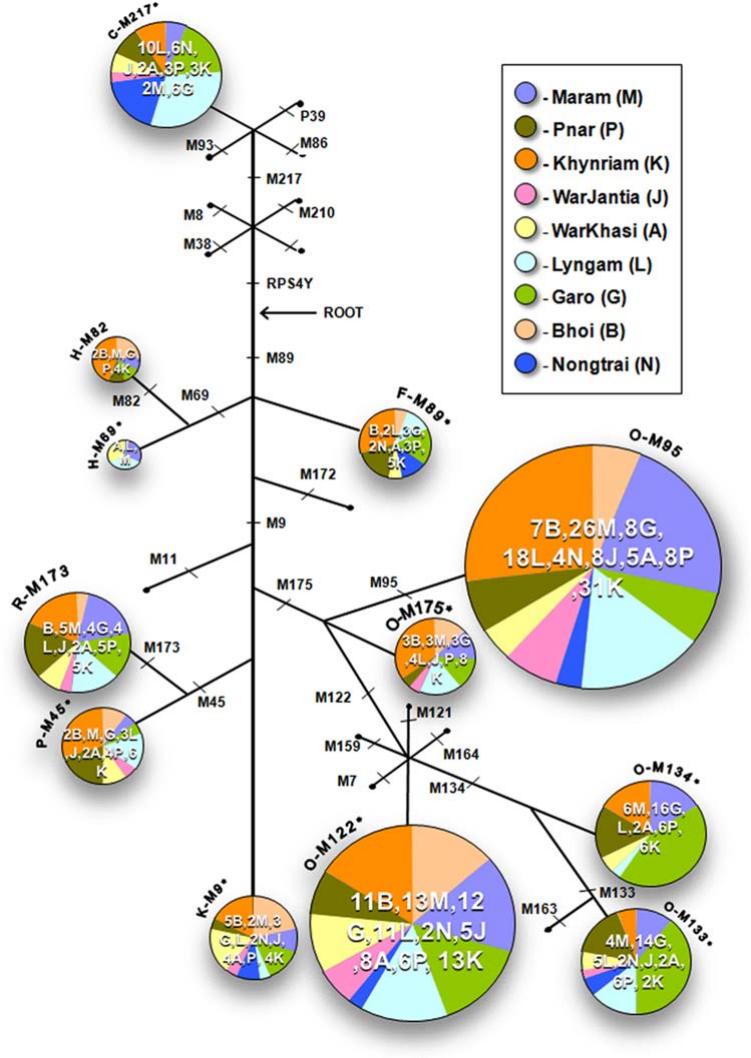
Rooted maximum-parsimony tree of Y-chromosome haplogroups defined by binary markers along with their frequency in Nine Meghalayan Populations.

Nei's [14] diversity statistic, *h,* based on the frequency of different haplogroups (Table 2), ranges from 77% in the Maram to 86.2% in the Pnar among the Khasi-Khmuic Austro-Asiatic groups, whereas it is 77.5% in the Tibeto-Burman Garo. For Y-STR haplotypes, while it ranges from 96.1% in Nongtrai to 99.9% in Khynriam in the Khasi-Khmic populations, it is 99.3% for the Garo. Out of the 26 potential haplogroups defined by the markers used in this study a total of 12 haplogroups were found in these populations (Figure 2). O-M95, with its frequency ranging from 17% in War-Khasi to 42% in War-Jaintia, was the most common haplogroup in all the Austro-Asiatic populations followed by the undifferentiated O-M122 (ranging from 11% in Nongtrai to 34% in Bhoi) where as in the Tibeto-Burman Garo the frequency of O-M134 and undifferentiated O-M122 haplogroups (23% and 17%, respectively) were the most common. H-M69 and its subclade H-M82 which is reported to be in high frequency in most of the Indo-European populations [15] are present with an average frequency of only 3% among them.

**Table 2:** Genetic Diversity (in percentage) based on Y-Chromosome and mtDNA analysis of populations from Meghalaya

Populations	Y-haplogroups	Y-haplotypes	mtDNa haplogroups	mtDNA haplotypes
Bhoi	81.7+/−4.3	99.6+/−0.9	93.6+/−2.3	95.3+/−2.0
Maram	77.0+/−3.7	99.6+/−0.4	89.4+/−2.3	92.8+/−2.5
Lyngngam	84.0+/−2.6	99.7+/−0.4	86.7+/−2.0	89.8+/−2.2
Nongtrai	83.7+/−5.5	96.1+/−3.4	87.8+/−2.7	92.0+/−3.1
War-Jaintia	77.8+/−7.8	99.1+/−1.8	83.1+/−5.1	86.8+/−5.5
War-Khasi	87.0+/−3.5	99.2+/−1.5	87.0+/−4.4	91.9+/−3.4
Pnar	86.2+/−2.7	99.2+/−0.8	89.7+/−2.0	95.4+/−1.5
Khynriam	82.7+/−2.9	99.9+/−0.2	90.9+/−1.6	96.1+/−1.1
*Average*	*82.5*	*99.0*	*88.5*	*92.5*
Garo	77.5+/−4.0	99.3+/−0.4	66.9+/−5.6	68.1+/−5.8

### Population structure based on Y-chromosome

**Figure 3:**
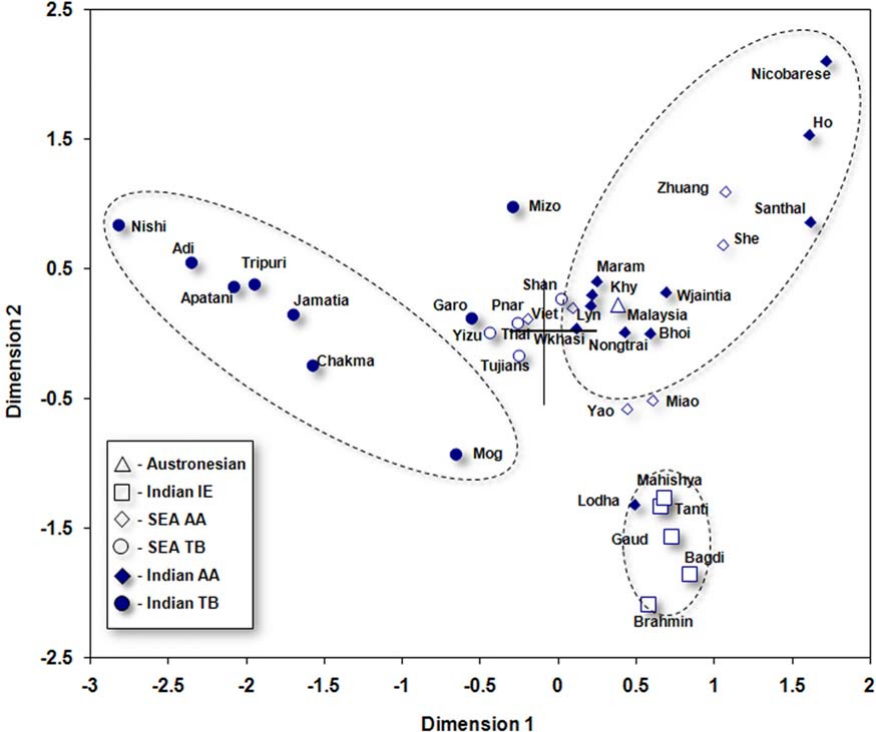
Plot on the first two dimensions derived from the multidimensional scaling of the pairwise F_ST_ distances of the populations based on Y-haplogroups. Reference to comparative data is given at Table 5. SEA, Southeast Asian; AA, Austro-Asiatic; IE, Indo-European, TB, Tibeto-Burman; Khy, Khynriam; Lyn, Lyngngam; Wkhasi, War Khasi; Viet, Vietnamese;

Based on the multidimensional scaling (MDS) of the Pairwise F_ST_ distances computed using haplogroup frequencies of Austro-Asiatic (Khasi from northeast India and others) and neighboring non-Austro-Asiatic populations, the two-dimensional MDS plot is furnished in Figure 3. A good fit between the two-dimensional MDS plot and the source data (pairwise value of F_ST_) was obtained (stress value of 18%). Broadly speaking, most of the Austro-Asiatic populations, including all the three linguistic sub-families of Austro-Asiatics i.e Mundari, Khasi-Khmuic and Mon-Khmer tribes, irrespective of their geographic affiliations, are placed in the upper right quadrant; Nicobarese, Ho, Santhal, She and Zhuang are somewhat removed from the others. On the other hand, most of the Tibeto-Burman populations are differentiated from the Austro-Asiatic populations and the Indo-European populations (clustered in the lower right quadrant) on the 1^st^ and 2^nd^ dimension, respectively. The Khasi-Khmuic populations, which form a compact cluster near the centroid, do not cluster with the Tibeto-Burman populations of Northeast India, barring the Garo of Meghalaya which has contiguous geographic distribution and marital interaction with them. Overall, the populations of the same linguistic family seem to cluster together, with few exceptions such as the Austro-Asiatic Lodha, which is placed among the Indo-European populations.

The analysis of molecular variance (AMOVA) yielded a significant but low F_ST_ values for both Y-SNPs (0.02) and STRs (0.02), suggesting a probable recent differentiation of the Khasi-Khmuic populations (Table 3). For Y-SNPs, whereas among group differentiation between the Khasi and Southeast Asian Austro-Asiatic populations is low (0.03) and non-significant it is relatively high and significant between the Khasi and Mundari populations (0.08). On the other hand, the F_CT_ value between Khasi-Khmuic and Indian Tibeto-Burman populations is very high and significant (0.30) while between Khasi-Khmuic and Southeast Asian Tibeto-Burman it was relatively low and non-significant (0.03). Although there is virtually no difference in the haplogroupic composition of the Tibeto-Burman Garo from Meghalaya and Southeast Asian Tibeto-Burman populations as suggested by the F_CT_ (−0.01627), it is surprisingly high (0.17975) between the Garo and the other Indian Tibeto-Burman populations.

**Table 3:** Analysis of Molecular Variance using Y-SNPs/STRs between groups of populations categorized on the basis of geography and languages

Groups	F_ST_	F_SC_	F_CT_
Khasi-Khmuic*	0.02		
Khasi-Khmuic Vs Garo*	0.04	0.02	**0.02**
Khasi-Khmuic	0.02		
Khasi-Khmuic Vs Garo	0.07	0.02	0.06
Khasi-Khmuic Vs SEA^1^-Tibeto-Burman	0.07	0.03	0.03
Khasi-Khmuic Vs Indian-Tibeto-Burman	0.32	0.02	0.30
Khasi-Khmuic Vs SEA- Austro-Asiatics	0.08	0.05	**0.03**
Khasi-Khmuic Vs Mundari	0.13	0.05	0.08
Garo Vs SEA-Tibeto-Burman	0.05	0.07	**−0.02**
Garo Vs Indian-Tibeto-Burman	0.17	−0.01	0.18

* Y-STR based analysis;

1 SEA, Southeast Asia; All values which are not in bold are significant at p<0.05

### Profile of new mtDNA haplogroups

**Figure 4:**
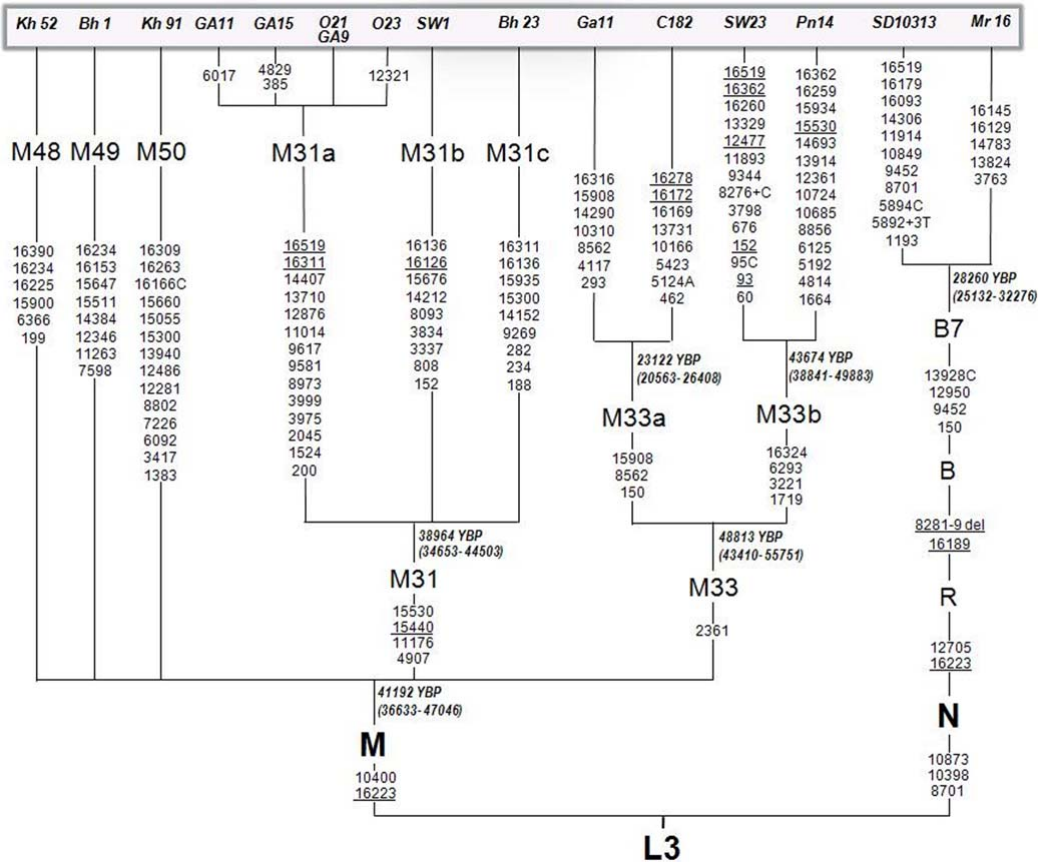
Phylogenetic tree of new haplogroups based on full mtDNA along with the TMRCA and associated 95% Confidence Interval. The samples names are on the tip of the haplogroups. The comparative data, for which sample names are written in capital letters, are from Kong et al. [17], Thangaraj et al. [3], Sun et al. [16] and Palanichamy et al. [19].

Based on Hypervariable segment (HVS) I and the known coding region SNPs most of the individuals could be assigned to specific haplogroups/lineages. However, there were still many individuals who could not be assigned to any existing lineages. Based on their HVS-I motif we could group these samples into 6 broad clades, and resequenced complete mtDNA of 1-2 samples from each of those clades to assign them to a known or new haplogroups (Fig 4). We also resequenced complete mtDNA for the samples falling in haplogroup B as none of the defining mutations for the subhaplogroups of B were found. The analysis of complete mtDNA suggests the presence of four new haplogroups which we have designated as M48, M49, M50 and B7. All the motifs in the coding region of the M48, except for 6336, which defines M30a [16] have not been reported and therefore we assign all these samples a new lineage. While the average frequency of M48 is 11% among the Austro-Asiatic Khasi groups, ranging from zero in War-Jaintia to as high as 26% in Lyngngam, it is present with a frequency of 4% among the Garo. Although haplogroups M49 and M50 are found with an average frequency of about 3% each in the Khasi populations, they could not be traced in the Garo as well as in some of the subgroups of Khasi. A subset of mutations at 150-9452-12950-13928C of our B-haplogroup samples has been reported in one of the samples (SD10313) of Han Chinese [17] which also falls in undifferentiated haplogroup B. We have proposed to name it as haplogroup B7 including the Han Chinese samples.

In addition to these four new haplogroups we propose two new sub-haplogroups –M33b- within M33, and M31c within M31 (Fig 4). The samples falling in M31c has all the defining mutations of M31 but do not share any of the coding region motifs with either M31a which has been reported in the Andamanese of Andaman and Nicobar island [3] and other Tribal populations of India [18], or M31b found in Rajbanshis (SW1) of Northeast India [19]. Therefore, we propose a new haplogroup, i.e. M31c. While this haplogroup is absent in the Garo, it is found with an average frequency of ∼5% in the Austro-Asiatic Khasi populations with a maximum frequency of ∼17% among the Bhoi. The samples of M33b have mutations which define M33 and it also shares mutations at positions 1719-3221-16293-16324 with the Rajbanshi sample (SW23) which is now re-designated as M33b. The frequency of M33b, with the exception of Pnar (∼22%) is low and found only in Lyngngam, Khynriam and Garo (∼2, ∼3 and ∼3%, respectively). On the other hand, M33a which were found to be in extremely high frequency in the Garo (∼55%) and with an average frequency of∼5% in Khasi-Khumic populations has been also reported in the Brahmins of Uttar Pradesh, India [16] and in the two populations of South India [20]. It is interesting to note that all the samples of this study, except one Khynriam sample, forms a single sublineage defined by 16316 HVS-I motif which distinguishes it from other M33a lineages found in other parts of India.

### Distribution and diversity of mtDNA haplotypes/lineages

**Figure 5:**
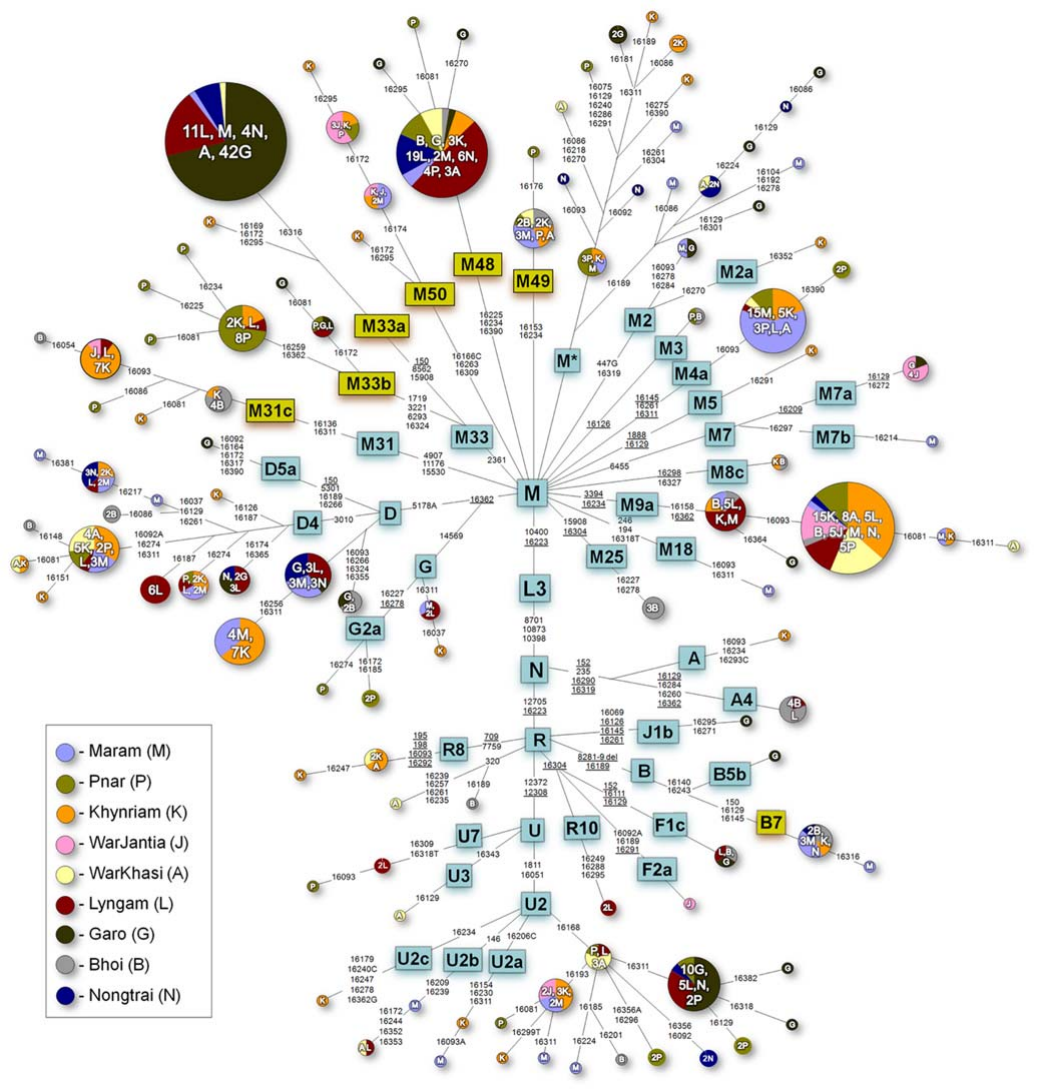
Tree Drawn from a Median-Joining Network of mtDNA Haplogroups Observed in Nine Meghalayan Populations. The haplogroups box in yellow colour are either new or redefined haplogroups.

In the 444 samples representing the 8 Khasi-Khmuic Austro-Asiatic tribes and a Tibeto-Burman Garo a total of 117 distinct HVSI haplotypes were found. Among these, 67 haplotypes are unique, each represented by single individual. Of the remaining, 37 are shared at least by two different tribes out of which only 10 are shared between Garo and Khasi subtribes. Based on the phylogenetic analysis of mtDNA control and coding region SNPs, 37 distinct haplogroups and subhaplogroups were observed among the studied populations (Fig 5). The samples that still remained unclassified in M and R are only ∼6%, and 0.5%, respectively. Among the Austro-Asiatic Khasi, ∼80% of the variation is accounted for by a set of 10 haplogroups–M*, M4a, M9a, M31c, M33a, M33b, M48, MD, MD4 and U2, whereas in the Garo a subset of only 3 haplogroups–M*, M33a and U2-accounted for ∼80% of the total sample. However, these 3 haplogroups account for only ∼18% of the sampled individuals from the neighbouring Austro-Asiatic Khasi populations.

The mtDNA haplogroup diversity (Table 2) among the Austro-Asiatic groups is low and ranges from 83.1% in War-Jaintia to 93.6% in Bhoi whereas in Garo the diversity is extremely low (66.9%). Similarly, the haplotype diversity (Table 2) for the Austro-Asiatic groups ranges from 86.8% in War-Jaintia to 96.1% in Khynriam where as in the Garo it is 68.1%.

### Population relationships based on mtDNA haplogroups

**Figure 6:**
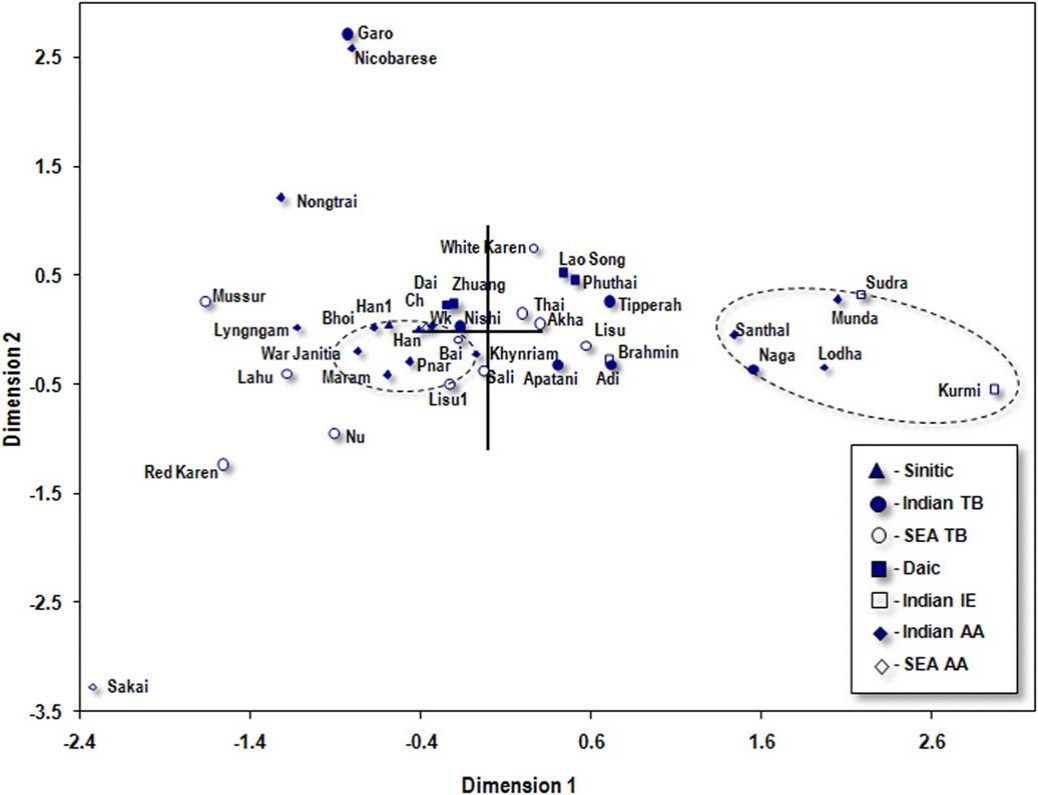
Plot on the first two dimensions derived from the multidimensional scaling of the pairwise F_ST_ distances of the populations based on mtDNA haplogroups. Reference to comparative data is given at Table 6. SEA, Southeast Asian; AA, Austro-Asiatic; IE, Indo-European; TB, Tibeto-Burman; Wk, WarKhasi; Ch, Chong;

The two dimensional plot of the multidimensional scaling of the genetic distance matrix of the 40 populations, including 8 Khasi subtribes and Garo of the present study and other relevant populations from the South and southeast Asia, is shown in Figure 6. The plot depicts the Tibeto-Burman Garo and Austro-Asiatic Nicobarese (a Mon-Khmer population) and Sakai as extreme outliers. As expected, the Mundari Austro-Asiatic populations, with predominantly South Asian mtDNA haplogroups, are placed as outliers aligning with the two Indian Indo-European populations on the extreme right corner of the plot. Although the Khasi-Khmuic Austro-Asiatic populations, except for Nongtrai, Lyngngam, form a constellation near to the left of centroid, it also has other populations such as Han, Lisu and Bai as part of this constellation. The Southeast Asian Tibeto-Burman populations is scattered along the 1^st^ axis. Similarly, the Indian Tibeto-Burmans do not form its own cluster. Overall, the three different sub-families of Austro-Asiatic populations do not form a homogeneous cluster, unlike in the case of Y-chromosome.

Although the AMOVA suggests low F_ST_ value (0.05), hence low differentiation among the Khasi-Khmuic populations (Table 4), it is quite high between them and the Garo (0.12). The differentiation of Khasi-Khmuic tribes with Southeast Asian Austro-Asiatic populations is moderate (0.05) but is much higher with those of Mundari populations (0.12). Surprisingly, the Tibeto-Burman Garo of Meghalaya shows high degree of differentiation with the other Tibeto-Burman populations of India (0.17) as well as Southeast Asia (0.13).

**Table 4:** Analysis of Molecular Variance using mtDNA haplogroups between groups of populations categorized on the basis of geography and languages

Groups	F_ST_	F_SC_	F_CT_
Khasi-Khmuic	0.05		
Khasi-Khmuic Vs Garo	0.16	0.05	0.12
Khasi-Khmuic Vs SEA^1^-Tibeto-Burman	0.10	0.07	0.04
Khasi-Khmuic Vs Indian-Tibeto-Burman	0.10	0.05	0.06
Khasi-Khmuic Vs SEA- Austro-Asiatics	0.11	0.06	0.05
Khasi-Khmuic Vs Mundari	0.17	0.05	0.12
Garo Vs SEA-Tibeto-Burman	0.20	0.08	0.13
Garo Vs Indian-Tibeto-Burman	0.21	0.05	0.17

1 SEA, Southeast Asia; All values are significant at p<0.05

### Time to Most Recent Common Ancestors (TMRCA)

The TMRCA was calculated based on mtDNA coding region (nucleotide position 577-16023) with the average sequence evolution rate as 1.26±0.08×10^−8^ base substitutions per nucleotide per year [21]. The TMRCA of the haplogroups based on the full mtDNA sequence (Fig 4) suggest a younger age of Khasi/Northeast Indian haplogroup M (41,000 YBP) compared to what has been obtained in the other studies [16] for Indian M haplogroup (54,000 YBP). This is because of the very low age contribution from the M48 haplogroup. Reanalyzing the data by removing M48 increases the age to ∼50,000 YBP which is close to what has been obtained in the other studies. The TMRCA of haplogroup M31 and M33 is ∼40,000 YBP and ∼50,000 YBP suggesting that M33 like M31 is an archaic lineage. The age of B7 suggests that this haplogroup has originated ∼28,000 YBP in East Asia where all the other sub-haplogroups of B have been hypothesized to have originated.

## Discussion

### Origin and spread of predominant Y-haplogroups of Northeast India

The present study provides a comprehensive genetic analysis of the populations of Austro-Asiatic linguistic family inhabiting the Northeast Indian region, which has probably served as the corridor for the peopling of Southeast Asia. Two major haplogroups O-M95 and O-M122 and its subhaplogroups dominate the haplogroup composition of the Austro-Asiatic and Tibeto-Burman populations of Northeast India and East and Southeast Asia (Table 5). Indo-European groups of Northeast India lack these haplogroups. Kumar et al. [22] suggest that the haplogroup O-M95 had its origin probably in the ancestors of Mundari, one of the major subfamilies of the Indian Austro-Asiatics inhabiting Eastern and Central regions of the country, and correlated its spread with the movement of these populations to Southeast Asia and to other parts inhabited by the Austro-Asiatics via the Northeast Indian corridor. Our results are consistent with this observation as we not only find relatively high frequency O-M95 (Table 5) in all the subgroups of Khasi from Northeast India, but also find a decreasing gradient of O-M95 average frequency from Mundari (53%) to Khasi (∼31%) to Southeast Asian Austro-Asiatics (∼23%), suggesting diffusion of O-M95 from India to Southeast Asia. Further, O-M95 is either absent or, if present, only with negligible frequency in the other linguistic groups of India. It is found to be either absent or has a negligible presence in the other Tibeto-Burman populations of Northeast India and the moderate frequency of O-M95 in the Tibeto-Burman Garo may be because of the gene flow from the neighboring Khasi which is facilitated by the practice of matrilocality in these populations. This suggests that the haplogroup O-M95 is, by and large, restricted to Austro-Asiatic populations in India and supports the contention of Kumar et al. [22] that probably there was a concomitant spread of the ancestors of the present day AA people with proto-Austro-Asiatic language and haplogroup O-M95.

**Table 5:** Y-haplogroup frequencies in different linguistic populations of South and Southeast Asia.

Haplogroups	Austro-Asiatic (Khasi-Khmuic)**	Austro-Asiatic (Mundari)	Nicobarese (Mon-Khmer)	Austro-Asiatic (Southeast Asia)	Garo (Tibeto-Burman)**	Tibeto-Burman (India)	Tibeto-Burman (East Asia)	Indo-European (Eastern India)
N (No. of populations)	353 (8)	64 (3)	11 (1)	257 (5)	71 (1)	226 (8)	214 (4)	54 (5)
C-RPS4Y	0.0	1.6	0.0	5.1	0.0	0.4	1.4	1.9
C-M217*	7.7	0.0	0.0	2.0	8.5	0.0	8.4	0.0
DE-YAP	0.0	0.0	0.0	2.7	0.0	0.9	4.2	0.0
F-M89*(xM69, M172)	4.0	10.9	0.0	1.2	4.2	0.0	0.9	5.6
H-M69	3.1	23.4	0.0	0.0	1.4	3.1	0.0	35.2
K-M9*(xM11, M45, M175)	5.7	0.0	0.0	0.8	4.2	1.3	13.1	0.0
O-M122*	19.6	0.0	0.0	42.8	16.9	1.8	23.4	1.9
O-M134*	12.2	0.0	0.0	10.1	42.3	79.2	24.3	0.0
O-M175*(xM95, M122)	5.7	0.0	0.0	10.9	4.2	0.0	13.1	0.0
O-M95	30.3	53.1	100.0	22.6	11.3	7.1	10.3	3.7
P-M45*(xM173)	5.4	10.9	0.0	1.6	1.4	3.1	0.0	18.5
R-M173	6.5	0.0	0.0	0.4	5.6	3.1	0.9	33.3

** Populations of this study; please note that some of the haplogroups were clubbed to have uniformity across different categories of populations

Comparative data are from Cordaux et al. [6], Su et al. [7], Sengupta et al. [15], Thangaraj et al. [37], Karafet et al. [38]

Haplogroup O-M122 is found to be in high frequency in the Garo as well as in Khasi-Khmuic populations. However, further typing of O-M122 chromosomes suggests a high frequency of undifferentiated O-M122 among the Khasi-Khmic populations, whereas the frequency of O-M134 (one of the subhaplogroups of O-M122) is found to be much higher among the Garo. Incidentally, O-M134 is found to be in much higher frequency compared to the undifferentiated O-M122 in the other Indian Tibeto-Burman populations as well. Further, Tibeto-Burman populations of Southeast Asia also have relatively much higher frequency O-M134 compared to the Austro-Asiatics there whose undifferentiated O-M122 samples fall mostly into subhaplogroup O-M159 (Table 5). The presence of O-M134 in high frequency among the Tibeto-Burman populations, both from India and East/southeast Asia, strongly suggests possibility of its correlation with the migration and spread of Tibeto-Burman populations into India.

### Distinct origin of Khasi-Khmuic tribes

Khasi-Khumic groups are surrounded by the predominant but ethnically similar Tibeto-Burman populations in the region and a comparison with them, particularly with the Garo which is also matrilocal like the Khasi and with whom they have contiguous distribution in Meghalaya, suggests a distinct genetic origin of the Khasi-Khmuic populations. For example, unlike the Khasi, the Tibeto-Burman Garo shows a very low frequency of O-M95 and undifferentiated O-M122 Y-haplogroups but a high frequency of differentiated O-M122 i.e. O-M134 and O-M133. Similarly, the other Tibeto-Burman groups from India show a low frequency of O-M95 and undifferentiated O-M122 but a very high frequency of O-M134 (Table 5). Further, while the Garo shows a high frequency of M33a (55%), it lacks M9a, MD and MD4 mtDNA haplogroups which are in high frequency among the Khasi. The other Tibeto-Burman groups from this region, although has 30% of unclassified M*, show a high presence F and its subhaplogroup, M8c, A and its subhaplogroups most of which have a negligible presence in the Austro-Asiatic Khasi populations. Moreover, M9a, MD and MD4 mtDNA haplogroups which account for ∼30% of Khasi-Khmuic samples are found only with a frequency of ∼10% among the Tibeto-Burman groups. Most importantly, Khasi-khumic group has ∼25% new haplogroups (M48, M49, M50, M31c and M33b) which has not been reported so far from any of the Northeast Indian groups except Garo which has M48 and M33b with a combined frequency of ∼7%. Thus, the composition of both the mtDNA and Y haplogroups in the Austro-Asiatic Khasi as a whole suggests their distinct origin and a separate migration vis-à-vis the Tibeto-Burman groups of this region.

### mtDNA landscape of the Meghalaya populations

The Khasi-Khmuic and the Garo populations of Meghalaya essentially have three kinds of haplogroups: the commonly found South Asian haplogroups, East Asian haplogroups and new haplogroups (Table 6 and Fig 5). Among the new haplogroups, B7 is found only in Khasi-Khmuic populations and it has been otherwise reported as unclassified B in Han Chinese [17] suggesting its probable origin in East Asia, as is the case with other haplogroups of B. However, all the other new haplogroups viz. M48, M49 and M50 or the redefined subhaplogroups i.e. M31c and M33b have not yet been reported from East Asia and the neighboring East Asian populations and these haplogroups needs to be assessed, especially from Myanmar region, to trace their origin and movement. Further, these haplogroups have not been reported among the 23 Indo-European and 25 Dravidian populations of the Indian subcontinent [23]–[25] confirming the absence of this haplogroups in other parts of India.

**Table 6:** mtDNA haplogroup frequencies in different linguistic populations of South and Southeast Asia.

Haplogroups	Austro-Asiatic (Khasi-Khmuic)**	Austro-Asiatic (Mundari)	Nicobarese (Mon-Khmer)	Austro-Asiatic (Southeast Asia)	Garo (Tibeto-Burman)**	Tibeto-Burman (India)	Tibeto-Burman (East Asia)	Indo-European (Eastern India)
N (No of Populations)	368 (8)	90 (3)	46 (1)	45 (2)	76 (1)	186 (5)	585 (11)	105 (3)
M*	5.4	42.2	8.7	11.1	6.6	30.1	17.8	53.3
South Asian^1^	21.5	56.7	0.0	0.0	17.1	5.9	5.5	39.1
East Asian^2^	42.4	0.0	91.3	88.9	13.2	64.0	76.8	1.9
West Asian^3^	0.8	1.1	0.0	0.0	1.3	0.0	0.0	5.7
New^4^	25.0	-	-	-	6.6	-	-	-
M33a	4.89	-	-	-	55.26	-	-	-

** This study; ^1^South Asian Haplogroups (HG) include M*, M2a, M2b, M3, M3a, M4a, M6, M6b, M10, M18, M25, R, R5, R6, R8, U2, U2abc, U3 and U4 ; ^2^East Asian HGs include A, B, F, M7, M8, M9 MG, MG and their subhaplogroups, N9a, W, X, Y, R9a and R10; ^3^West Asian HGs include U7, H, J1 and T; ^4^New M (sub)haplogroups are M33b, M48, M49 and M50, and M31c which are confined in the region of Northeast India and need to be typed in comparative populations for its status.

Comparative data are from Fucharoen et al. [39], Oota et al. [40], Prasad et al. [41], Roychoudhury et al. [23], Yao et al. [42]–[43], Metspalu et al. [25]

The phylogeny of M33 and M31 is quite intriguing. It is striking that these two lineages have evolved into ethnic specific branches, separated by a number of mutations, suggesting their deep antiquity. Haplogroup M31 has three subhpalogroups-M31a, M31b and M31c (Fig 4). While M31a is reported with a high frequency in the Greater Andmanese [3] and Lodha, Chenchu and Lambadi tribal groups of India [18], M31b and M31c are found in Northeast India, M31b among the Rajbanshis [19] and M31c with an average frequency of ∼5% in the Khasi-Khmuic populations (Fig 5). However, this haplogroup is absent in the Tibeto-Burmans of Northeast India or among the other Indian populations. Haplogroup M33b has a total frequency of ∼4% among the Khasi and otherwise it has been reported only from Rajbanshi from this region. On the other hand, haplogroup M33a, which has been reported in the Brahmins of Uttar Pradesh, India [16] and in the two populations of South India [20] bifurcates into two branches-one, without the motif 16316, identified in only 1 Khynriam sample and another defined by 16316 HVS-I motif and found in all the samples of M33a of Garo (∼55%) and Khasi-Khumic populations of Meghalaya (∼5%). However, none of the other Indian Austro-Asiatic populations (Mundari) shows M33 or its subhaplogroup (Kumar et al., unpublished results). Although we need to screen more populations in order to resolve the origin and distribution of M31 and M33, both these lineages are very old (>40,000 YBP) and probably originated in mainland India as their presence has been reported only from the Indian subcontinent. Overall, the new and the redefined haplogroups, excluding M33a, account for ∼25% of the total frequency in the Khasi-Khmuic populations and ∼4% in the Garo, while M33a accounts for ∼55% in Garo. The rest of the haplogroups are of either South Asian or East Asian types.

The East Asian haplogroups with high frequency in the Khasi-Khmuic populations (M9a, MD and MD4) account for ∼35% of the samples. Along with B7, the presence of very different set of East Asian haplogroups among them, as compared to the Tibeto-Burmans of India, may suggest a strong possibility of their admixture with or assimilation of certain East Asian populations, other than the Indian Tibeto-Burmans. This is also reflected in case of Y-chromosome with high frequency of undifferentiated O-M122. The South Asian haplogroups found in Khasi-Khmuic populations are M2 and M2a, M18, M25, M3, M4a, M5, R, R8, U2 and U2abc, and U3 which account for a total of ∼22% among them. However, the frequency of all these haplogroups is quite low except for M4a and U2, which account for ∼7 and ∼8%, respectively. The low frequency of South Asian haplogroups might be either due to admixture with the surrounding Indo-European populations or due to initial splintering of their gene pool from that of the Mundari tribes as both these linguistic groups show predominantly South Asian mtDNA haplogroups.

### The Austro-Asiatic tribes of Northeast India: Genetic link/continuity between South and Southeast Asian populations

A rapid human migration through Southern route ∼60,000 YBP is suggested to have brought undifferentiated M and R into South and East Asia and subsequently differentiated into different subhaplogroups in different regions [17]. For example, the undifferentiated M evolved into different haplogroups such as M2, M3, M5 M6, M18 etc. in South Asia, whereas in East Asia, primarily haplogroup M9, MD, MG etc. evolved. In this backdrop, it is interesting that the Mundari Austro-Asiatic tribes of central and eastern India have mostly South Asian haplogroups (Kumar et al. unpublished results) with a high frequency of M2b, M40a, R6 and R7, which are considered to be the old lineages. This may support the migration of these tribes into India by Southern route and using the Western Indian corridor, bringing with them the undifferentiated M and R ∼50,000 YBP. The predominant lineages found among them might have evolved subsequently. However, these mtDNA haplogroups are with very low frequencies in the Khasi-Khmuic Austro-Asiatic populations from Northeast India who, nonetheless, have ∼25% of their haplogroups as new ones (Table 6). This would be feasible only if a section of them had separated soon after the ancestors of Austro-Asiatics had come to India, and migrated quite rapidly to Northeast India and further to Southeast Asia before the differentiation of M and R into any of the haplogroups found in South Asia. The undifferentiated M and R haplogroups carried by this group might have evolved into certain characteristic haplogroups such as M48, M49 and M50 in the Khasi but not in the Mundari groups around 40,000 YBP. A section of these initial migrants to Northeast India had probably moved further to Southeast Asia in rapid succession carrying with them the undifferentiated M (before the Khasi- specific haplogroups could evolve), which might have evolved into typical East Asian haplogroups. It is evident from the foregoing discussion that the Austro-Asiatic Khasi of Northeast India represents genetic continuity, linking the populations of South and Southeast Asia. Therefore, our findings reinforce the suggestion that Northeast India has acted as a corridor for initial movement of populations, not as a barrier as suggested in a recent study [6].

## Materials and Methods

### Collection of blood samples

About 5 ml of intravenous blood samples were collected in 5 ml Tarson tubes containing EDTA as an anticoagulant from a total of 444 healthy unrelated volunteers, both males and females, after obtaining the informed written consent. Prior approval for the study was obtained from the ‘Indian Statistical Institute Review Committee for Protection of Research Risk to Humans’. These samples represent 8 subgroups of the Austro-Asiatic Khasi, besides the neighbouring Tibeto-Burman Garo tribes from Meghalaya in the Northeastern part of India (Table 1 and Figure 1). While the samples for the different dialectical groups of Khasi tribe were drawn from almost all the areas of their distribution in Khasi hills, Garo samples were represented in bulk from the contiguous areas of the Lyngngam tribe, such as Rongjeng, Khonjoy, Shallang, etc. with a few samples drawn from all over the capital city of Meghalaya, Shillong.

### mtDNA typing

The mtDNA genomes were amplified and sequenced by means of the procedures described in a recent study [17]. Sequences were edited and mutations scored relative to the revised Cambridge Reference Sequence [26] (rCRS). Initially, HVS-I (nucleotide positions [nps] 160001–16400) was sequenced, besides typing SNPs at 10398 and 10400. The published HVS-I sequences [16], [24], [27]–[30] were compared with our HVS-I sequences from Meghalaya in combination with the typed SNPs of this study to identify the relevant coding region SNPs, which are diagnostic of the main haplogroups and subhaplogroups within the mtDNA phylogeny. These coding regions were then selectively assayed by sequencing to obtain haplogroups and their derivatives (Fig 5). Representative samples from those that could not be assigned into known haplogroups were carefully chosen and complete mtDNA sequencing was done to assign/designate them to the new (sub)haplogroups (Fig 4).

### Y-chromosome typing

The following 25 Y-SNPs which are known to detect variations in Asia were screened: RPS4Y, M210, M38, M8, M217, M93, M86, M89, M69, M82, M172, M9, M175, M122, M7, M164, M159, M121, M134, M133, M162, M95, M11, M45 and M173 [31]–[33]. The rooted maximum-parsimony trees of the haplogroups defined by these markers are presented in Figure 2. Many of the samples were typed with all the binary markers for internal check on the reliability of the typing and also to detect recurrent mutations. The nomenclature as suggested and followed by Y-chromosome consortium [32] was used. The following 6 Y-STRs loci were also typed: DYS19, DYS389I, DYS389b, DYS390, DYS391 and DYS393 (for data refer to Dataset S1) and the details of these loci are given at Butler et al [34].

### Statistical Analysis

Since the DYS389II allele length also contains DYS389I, for all statistical analyses a simple subtraction of DYS389I allele length from that of DYS389II was done to avoid the double-counting variation at DYS389I. The subtracted DYS389II allele is named as DYS389b. The Y-SNP and modified Y-STR data were then analyzed for haplogroup and haplotype diversity, respectively, along with their associated Standard Error by means of the software package ARLEQUIN 3.01 [35]. The frequencies of haplogroups constructed by binaray markers were used to compute pairwise F_ST_ genetic distance matrix. Based on the distance matrix, MDS analysis was performed using SPSS package. The genetic structure as reflected in the distribution of Y-SNPs and STRs was further explored through AMOVA, by grouping populations based on their geography and linguistic affiliations

Haplotype and Haplogroup diversity of mtDNA and AMOVA based on haplogroup frequencies were calculated using the ARLEQUIN 3.01 [35]. Phylogenetic relationships between the observed haplogroups were first drawn by hand and then confirmed by using the NETWORK program [36]. The frequencies of haplogroups were used as input vector to compute pairwise F_ST_ genetic distance matrix using Arlequin 3.01. Based on this distance matrix, MDS analysis was performed using SPSS package. The TMRCA of the clades and subclades and their associated SEs were calculated using their full mtDNA sequences [21].

## Supporting Information

Dataset S1 Six Y-STR data of all the 9 populations
(0.05 MB DOC)
