# Supplementary material for: Austro-Asiatic Tribes of Northeast India Provide Hitherto Missing Genetic Link between South and Southeast Asia
Source: PLoS One. 2007 Nov 7;2(11):e1141. doi: 10.1371/journal.pone.0001141 (PMC2065843; doi:10.1371/journal.pone.0001141)
Supplement: Supplementary file 1 [file pone.0001141.s001.doc]

## Sheet: Sheet1

Dataset S1: Six Y-STR data of all the 9 populations
	393	19	390	389I	391	389B
Bhoi
BH1	12	15	23	9	10	19
BH2	13	14	23	10	10	16
BH3	12	15	23	10	11	17
BH4	11	14	24	9	10	17
BH5	13	15	25	11	10	16
BH6	12	15	22	9	10	17
BH7	12	16	22	9	10	17
BH8	12	15	23	10	10	18
BH9	11	15	22	11	10	17
BH10	12	14	23	10	10	18
BH11	11	15	23	9	10	17
BH12	11	14	23	9	11	18
BH13	15	15	25	10	11	17
BH14	12	15	23	9	11	18
BH15	11	11	23	9	11	19
BH16	13	15	25	11	11	18
BH17
BH18	13	15	25	10	10	17
BH19	11	14	22	11	10	19
BH20	13	15	23	10	11	17
BH21	13	14	21	10	10	19
BH22	11	11	23	10	10	17
BH23	11	14	23	11	10	17
BH24	13	17	23	9	10	22
BH25	13	15	24	10	10	17
BH26	13	15	25	11	11	16
BH27	11	15	23	9	10	19
BH28	11	11	25	11	11	16
BH29	13	14	25	10	10	16
BH30	13	15	25	10	10	18
BH31	13	15	25	11	11	18
BH32
Maram
MR1	16	15	25	9	10	17
MR2	13	14	23	10	10	16
MR3	13	15	26	9	10	17
MR4	12	12	19	12	10	14
MR5	13	14	23	11	10	15
MR6	12	14	23	9	10	18
MR7	14	15	24	10	11	17
MR8	13	15	25	9	11	18
MR9	13	14	24	9	11	18
MR10	13	14	24	8	10	19
MR11	14	14	23	10	10	16
MR12	13	15	24	9	10	21
MR13	14	17	25	9	10	19
MR14	14	15	25	9	10	18
MR15	15	11	23	11	10	20
MR16	14	10	23	10	10	18
MR17	13	15	23	11	11	16
MR18	13	15	25	11	10	15
MR19	11	13	22	11	11	16
MR20	11	15	20	10	10	16
MR21	11	12	19	12	10	16
MR22	12	18	22	11	10	18
MR23	16	15	25	9	10	17
MR24	16	12	23	11	10	15
MR25	13	14	23	10	10	16
MR26	13	15	26	9	10	17
MR27						0
MR28	12	16	25	11	11	17
MR29
MR30
MR31	12	18	25	11	10	17
MR32	13	15	26	11	13	18
MR33	13	15	25	10	10	17
MR34	13	15	26	10	11	15
MR35	13	15	24	10	11	16
MR36	14	15	23	11	10	15
MR37	13	16	25	10	10	17
MR38	11	15	22	11	13	20
MR39	12	15	23	11	10	17
MR40	12	15	23	10	10	18
MR41	12	14	23	9	11	19
MR42	12	15	22	10	11	17
MR43	12	15	23	10	10	19
MR44	12	15	25	11	10	17
MR45	12	15	25	11	10	17
MR46	12	14	25	10	10	18
MR47	14	15	23	11	11	15
MR48	14	15	24	10	10	17
MR49	11	16	23	10	11	18
MR50	14	16	25	11	11	16
MR51	12	16	25	10	11	16
MR52	12	15	23	11	11	20
MR53	12	16	25	11	11	17
MR54	13	15	25	9	10	18
MR55	12	16	25	10	10	17
MR56	12	14	24	10	11	19
MR57	13	15	24	10	11	16
MR58	13	15	25	10	11	16
MR59
MR60	12	14	23	9	10	17
MR61	11	15	23	11	11	15
MR62	13	14	24	9	11	18
MR69
MR73
Lyngngam
LY1	11	14	23	11	10	16
LY2	12	16	25	9	10	17
LY3	13	15	23	11	10	16
LY4	13	14	24	9	10	16
LY5	13	14	24	10	10	15
LY6	13	15	24	10	10	17
LY7	13	15	23	10	10	17
LY8	12	16	25	9	10	17
LY9	12	15	25	10	10	17
LY10	12	15	24	9	10	18
LY11	13	14	23	9	10	18
LY12	13	15	25	8	10	21
LY13	13	15	24	9	10	18
LY14	12	15	23	8	10	19
LY15	12	12	23	9	10	17
LY16	13	15	25	9	10	17
LY17	13	15	25	9	10	19
LY18	11	15	23	9	10	17
LY19	12	15	24	14	10	14
LY20	14	12	24	9	10	19
LY21	16	13	23	9	10	17
LY22	13	16	24	9	11	18
LY23	11	14	23	9	10	16
LY24	12	15	23	9	11	18
LY25	12	15	23	9	11	17
LY26	13	15	24	9	11	18
LY27	13	15	24	8	10	19
LY28	11	15	23	9	10	18
LY29	11	15	24	9	10	17
LY30	12	17	24	10	10	17
LY31	13	14	23	9	10	17
LY32	13	15	24	9	10	18
LY33	11	15	23	9	10	17
LY34	12	15	25	10	11	16
LY35	13	15	25	11	11	16
LY36	13	15	25	10	11	16
LY37	12	15	25	11	10	18
LY38	12	15	25	10	10	19
LY39	12	13	24	10	10	17
LY40	12	16	25	11	11	17
LY41	13	16	24	10	10	18
LY42	16	15	23	10	11	17
LY43	12	15	23	10	11	17
LY44	13	15	24	9	10	18
LY45	13	15	25	11	11	15
LY46	14	15	25	10	10	17
LY47	13	15	25	10	11	16
LY48	11	15	25	9	10	17
LY49	12	16	25	10	10	17
LY50	13	16	25	9	10	17
LY51	12	16	25	10	10	16
LY52	14	16	25	10	10	16
LY53	14	15	23	10	10	17
LY54	11	17	24	9	10	17
LY55	13	17	24	10	10	17
LY56	13	15	23	10	11	16
LY57	13	15	26	10	10	14
LY84
LY85
LY86
Nongtrai
NO1	13	15	26	11	10	15
NO2	12	15	23	10	10	17
NO3	13	14	23	11	11	17
NO4	13	15	24	10	11	17
NO5	12	15	24	12	10	18
NO6	13	15	23	10	10	16
NO7	13	15	23	10	11	17
NO8	13	15	23	10	11	17
NO9	13	15	23	10	11	17
NO10	13	15	23	10	11	16
NO11	13	15	23	10	11	16
NO12	13	15	23	10	11	16
NO13	13	14	23	10	11	16
NO14	16	14	24	10	10	17
NO15	13	16	23	9	10	17
NO16	11	14	23	9	10	17
NO17	13	16	25	10	11	17
NO18	11	15	23	9	10	17
NO21
War Jaintia
WJ1	13	15	25	10	11	17
WJ2	10	14	27	11	11	18
WJ3	12	14	24	9	10	18
WJ4	13	16	22	9	9	19
WJ5	13	15	25	11	10	17
WJ6	13	16	23	10	10	17
WJ7	13	16	25	11	11	16
WJ8	13	16	25	11	11	17
WJ9	13	15	25	10	10	17
WJ10	11	15	24	10	10	18
WJ11	13	15	24	9	10	19
WJ12	12	16	25	10	10	16
WJ13
WJ14	12	16	25	9	11	19
WJ15	12	15	23	10	10	18
WJ16	12	15	23	10	10	17
WJ17	13	15	25	10	10	17
WJ18	13	14	25	10	11	17
WJ19	12	15	23	10	10	18
WJ20	13	15	25	10	10	18
War Khasi
WA1	12	15	25	10	11	17
WA2	13	15	26	10	10	17
WA3	13	15	24	9	10	19
WA4	14	15	23	10	10	16
WA5	14	15	23	9	10	17
WA6	14	15	23	10	10	16
WA7	13	16	26	11	11	15
WA8	12	15	23	11	11	19
WA9	13	15	25	11	11	15
WA10	14	15	25	10	9	15
WA11	12	15	23	11	11	17
WA12	12	15	23	10	10	16
WA13	13	15	25	10	10	16
WA14	13	15	25	10	11	16
WA15	14	15	23	10	10	15
WA16	14	15	23	9	10	17
WA17	12	15	23	10	10	13
WA18	12	16	25	11	11	18
WA19	12	14	26	10	10	18
WA20	12	15	25	10	11	20
WA21	12	15	23	10	11	18
WA22	13	15	25	11	10	17
WA23	11	16	23	9	10	18
WA24
WA25
WA26
WA27
WA28
WA29
Pnar
PN1	11	15	21	10	10	17
PN2	12	15	23	11	11	18
PN3	12	16	25	11	11	20
PN4	11	15	21	10	10	17
PN5	11	15	23	11	11	17
PN6	12	14	22	12	11	17
PN7	16	15	23	11	11	17
PN8	13	15	24	10	10	18
PN9	13	15	24	9	10	18
PN10	12	15	21	14	10	13
PN11	11	16	20	10	10	17
PN12	13	15	24	10	10	18
PN13	14	14	25	11	11	17
PN14	11	15	21	10	10	17
PN15	11	15	23	12	10	16
PN16	13	15	23	10	11	18
PN17	11	10	23	9	10	17
PN18	11	14	24	11	10	15
PN19	12	14	23	9	10	17
PN20	12	16	25	10	10	16
PN21	12	14	25	11	10	18
PN22	11	15	25	9	10	17
PN23	11	15	25	9	10	17
PN24	11	15	26	11	10	15
PN25	12	16	25	10	10	18
PN26	12	16	23	9	10	19
PN27	12	15	23	10	10	18
PN28	12	15	23	10	10	18
PN29	12	15	26	10	10	16
PN30	12	15	23	11	12	16
PN31	12	15	23	11	10	17
PN32	10	13	23	9	10	18
PN33	11	14	24	10	11	19
PN34	12	14	22	11	11	17
PN35	12	14	22	11	11	15
PN36	14	15	25	10	10	15
PN37	13	15	23	10	10	16
PN38	14	15	23	10	10	16
PN39	13	15	25	10	10	16
PN40	13	15	25	12	10	18
PN41
PN46
PN49
PN52
Khynriam
KH1	13	15	23	9	11	19
KH2	11	16	24	10	10	17
KH3	12	15	23	10	11	17
KH4	12	16	23	11	11	18
KH5	12	14	23	10	10	18
KH6	13	13	23	10	10	18
KH7	15	15	23	9	10	19
KH8	12	16	23	12	10	16
KH9	12	15	25	11	10	18
KH10	13	15	25	10	10	16
KH11	12	15	23	11	10	18
KH12	12	16	24	10	11	17
KH13
KH14	14	15	25	11	10	15
KH15	11	14	23	10	10	16
KH16	15	15	23	10	11	16
KH17	16	16	25	10	11	16
KH18	13	15	24	11	10	18
KH19	12	15	25	9	10	17
KH20	15	16	25	11	10	15
KH21	16	15	25	9	10	17
KH22	14	15	25	9	10	16
KH23	11	15	25	9	10	17
KH24	13	14	23	9	10	17
KH25	13	10	25	10	11	18
KH26	12	11	23	10	10	19
KH27	13	12	24	10	10	15
KH28	14	12	23	10	10	18
KH29	12	13	23	10	11	18
KH30	14	15	23	10	11	19
KH31	12	13	26	10	10	15
KH32
KH33	12	14	23	12	10	19
KH34	12	15	23	11	11	19
KH35	13	15	25	10	10	18
KH36
KH37
KH38	13	15	25	10	11	17
KH39	13	16	25	10	10	17
KH40	14	15	25	12	10	14
KH41	13	14	23	11	10	18
KH42	12	15	23	11	11	17
KH43	11	17	25	10	10	17
KH44	14	15	26	9	11	17
KH45	14	16	20	10	9	16
KH46	16	16	24	11	10	17
KH47	13	17	28	11	11	14
KH48	14	15	24	10	11	13
KH49	14	15	23	10	10	16
KH50	14	10	23	10	10	13
KH51	12	15	23	9	10	19
KH52	13	15	23	9	10	19
KH53	11	14	23	9	10	17
KH54	12	15	23	10	10	18
KH55	13	15	25	10	10	16
KH56	11	14	28	9	10	17
KH57	11	12	22	8	8	15
KH58	12	15	23	11	11	18
KH59	13	15	25	10	10	17
KH60	13	16	24	10	10	16
KH61	14	15	23	10	10	16
KH62	13	15	24	10	10	17
KH63	13	11	24	10	11	18
KH64	13	15	23	10	10	18
KH65	13	15	24	10	10	18
KH66	11	15	22	11	10	17
KH67	13	14	22	10	10	17
KH68
KH69	11	15	23	9	10	18
KH70	14	16	23	9	10	17
KH71	13	15	25	11	10	15
KH72	13	15	23	10	10	17
KH73	13	15	21	10	10	17
KH74	13	14	23	9	11	18
KH75	12	15	23	10	10	19
KH76	14	15	25	11	11	15
KH77	13	14	24	9	10	18
KH78	11	17	24	10	10	17
KH79	14	15	25	10	11	16
KH80	13	16	25	11	11	16
KH81	13	16	25	10	11	17
KH82	14	14	25	10	11	18
KH83	14	15	25	10	10	16
KH84	13	14	23	10	10	17
KH85	11	14	24	9	10	16
KH86	11	15	22	10	10	16
KH87	12	15	23	10	10	18
Garo
GA1	12	14	26	9	10	18
GA2	11	14	26	8	10	21
GA3	13	13	24	9	10	18
GA4	12	14	24	8	10	19
GA5	11	14	24	8	10	19
GA6	13	15	24	10	10	17
GA7	12	15	23	9	10	18
GA8	11	15	23	10	10	17
GA9	12	15	24	9	10	18
GA10	12	15	24	9	10	18
GA11	13	15	24	10	10	16
GA12	11	15	24	9	10	17
GA13	12	15	24	9	10	18
GA14	12	15	23	10	10	17
GA15	11	15	24	11	10	15
GA16	11	15	23	9	10	20
GA17	12	15	23	11	10	16
GA18	12	15	25	11	10	16
GA19	12	13	24	10	10	17
GA20	13	14	22	10	10	17
GA21	11	14	23	9	10	16
GA22	12	15	23	9	11	17
GA23	11	15	23	9	10	17
GA24	11	14	23	9	10	17
GA25	12	15	25	9	10	22
GA26	12	15	23	9	10	18
GA27	13	15	24	9	10	18
GA28	13	15	24	10	10	17
GA29	12	15	23	9	10	18
GA30	11	15	23	8	10	19
GA31	13	14	22	10	10	17
GA32	12	15	24	9	10	17
GA33	12	15	24	9	10	17
GA34	11	17	24	9	10	17
GA35	13	14	25	10	11	17
GA36
GA37	11	16	24	9	11	17
GA38
GA39
GA40
GA41
GA42
GA43	12	15	24	9	10	17
GA44
GA45
GA46
GA47	12	15	25	10	10	19
GA48	11	15	24	10	10	17
GA49
GA50	13	15	29	11	10	18
GA51	13	14	23	11	9	17
GA52	12	14	24	9	9	19
GA53	13	18	25	11	10	18
GA54	12	15	23	10	11	18
GA55	11	14	23	9	10	16
GA56	12	14	23	9	10	17
GA57	11	15	23	9	10	17
GA58	12	15	23	9	10	17
GA59	11	15	23	9	10	17
GA60	10	15	23	9	10	19
GA61	11	14	23	9	10	18
GA62	13	15	22	11	10	17
GA63	11	15	25	11	11	17
GA64	13	14	24	10	9	16
GA65	13	15	25	10	10	18
GA66	11	14	23	9	10	17
GA67	13	15	24	10	10	15
GA68	14	15	24	10	10	15
GA69	12	15	25	11	11	16
GA70
GA71	12	14	23	9	10	16
GA73
GA74
GA75
GA77	13	16	25	10	10	16
*The Y-SNPs and STRs data of the sample which are underlined and italicized are from Kumar et al. [22]
**The blank rows with only sample name have only Y-SNP data and its corresponding Y-STR data is not available

## Sheet: Sheet2

## Sheet: Sheet3
